# Dog exposure and subsequent asthma outcomes in children with asthma and allergy

**DOI:** 10.1016/j.jacig.2026.100692

**Published:** 2026-03-20

**Authors:** Resthie R. Putri, Cecilia Lundholm, Bronwyn K. Brew, Hanna Karim, Jon R. Konradsen, Tove Fall, Catarina Almqvist

**Affiliations:** a Department of Medical Epidemiology and Biostatistics, Karolinska Institutet, Stockholm, Sweden; c Paediatric Allergy and Pulmonology Unit, Astrid Lindgren Children's Hospital, Karolinska University Hospital, Stockholm, Sweden; d Molecular Epidemiology, Department of Medical Sciences, Uppsala University, Uppsala, Sweden; e Science for Life Laboratory, Uppsala University, Uppsala, Sweden; b School of Medicine and Public Health, University of Newcastle, Newcastle, Australia

**Keywords:** Asthma, allergy, allergic asthma, dog, dog exposure, asthma exacerbation, asthma severity

## Abstract

**Background:**

While early-life dog exposure and its association with subsequent asthma is well studied, less is known about the impact of continuous or discontinued exposure on asthma outcomes in children with established asthma and allergy.

**Objectives:**

We sought to estimate the association between dog exposure and long-term asthma outcomes.

**Methods:**

A cohort study was conducted using Swedish national registers, following 99,389 children aged 3-16 years at asthma and allergy diagnosis until age 19, emigration, death, or year 2023. Dog exposure was categorized as “continuous” (parental dog ownership at diagnosis and throughout follow-up), “discontinued” (ownership ceased sometime after diagnosis), and “no exposure.” Outcomes included moderate-to-severe asthma (defined by treatment steps) at 2-, 4-, and 6-year follow-up and asthma exacerbation (emergency visits and high short-acting β-2 agonist use) throughout follow-up.

**Results:**

In the cohort (median age 6.6 years; 41% female), 12.8% had continuous exposure and 1.2% had discontinued exposure. Compared to nonexposed, no association between continuous exposure and moderate-to-severe asthma was observed (adjusted odds ratio at 2-year follow-up: 1.00; 95% CI: 0.94-1.07); discontinued exposure showed similar result (adjusted odds ratio: 1.07; 95% CI: 0.93-1.23). However, both exposure groups had increased risk of exacerbations (continuous exposure: adjusted hazard ratio: 1.17; 95% CI: 1.06-1.29; and discontinued: hazard ratio: 1.52; 95% CI: 1.15-2.00), with no significant difference between the groups.

**Conclusion:**

In children with established asthma and allergy, continuous dog exposure does not seem to increase the risk of moderate-to-severe asthma, but it is associated with a modest increased risk of exacerbations. Discontinued exposure does not appear to improve asthma outcomes at the population level.

## Graphical abstract

**Figure:**
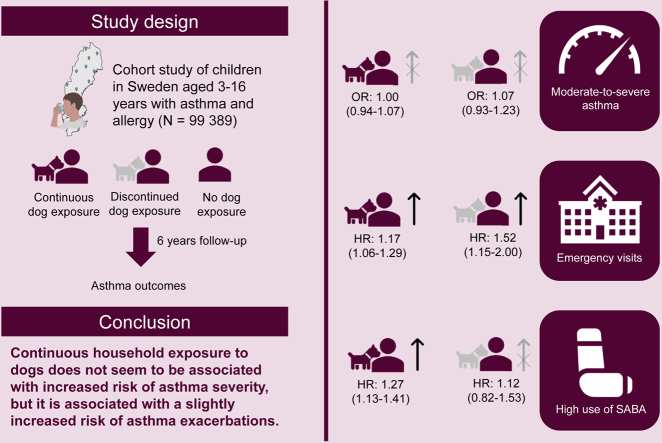

Asthma remains the most common chronic disease in children, with only one-half of them estimated to have well-controlled asthma.^1^ To improve asthma control in sensitized patients, environmental avoidance such as the removal of furry animals is commonly advised despite its unproven effectiveness.^2^, ^3^, ^4^ Companion animals, most frequently cats and dogs, are common in European households,^5^ and notably, asthma diagnosis does not seem to deter families from keeping or acquiring a dog.^6^^,^^7^

The relationship between exposure to dogs and pediatric asthma is intricate. Previous meta-analysis of multiple birth cohorts has shown that early pet ownership does not increase risk of asthma or allergy.^8^ Yet, sensitization to dog allergens is associated with an increased risk of developing asthma^9^ and risk of having severe asthma among children with asthma.^10^^,^^11^ However, the long-term impact of continuous household exposure to dogs on asthma outcomes in children with airway allergy remains unknown. Previous studies, limited by the cross-sectional design,^12^, ^13^, ^14^, ^15^ selected population,^13^^,^^14^^,^^16^ and self-reported data,^12^, ^13^, ^14^, ^15^, ^16^ showed inconsistent results (positive,^12^^,^^16^ inverse^13^ and null associations^14^^,^^15^). In clinical practice, families often ask whether having a dog influences future asthma outcomes and whether discontinuing dog exposure could help. These questions remain unanswered given the conflicting evidence and uncertainty about long-term effects.

Using nationwide data, this study aimed to assess:

• Among children with asthma and airway allergy, is continuous or discontinued household exposure to dogs following diagnosis associated with moderate-to-severe asthma at 2-, 4-, and 6-year follow-up, compared to no exposure?
• Is continuous or discontinued exposure to dogs associated with increased risk of asthma exacerbation during pediatric years compared to no exposure?

## Methods

### Study design and population

**Fig 1:**
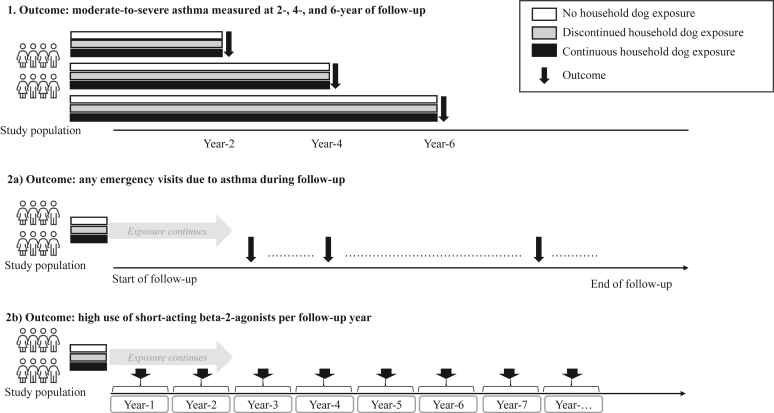
Study design. The baseline period was measured from the time of diagnosis to 364 days after the diagnosis, and the follow-up times begin 1 year after diagnosis.

A cohort study included all children born in Sweden (2002-2018) who, between ages 3 and 16 years, met validated criteria for both asthma and airway allergy,^17^, ^18^, ^19^ as a proxy for allergic asthma. Asthma was identified by either an asthma diagnosis in specialized care or 2 dispensations of asthma medications,^17^ while allergy was defined by a diagnosis and/or medications for allergic rhinoconjunctivitis^18^ (see diagnosis and medicine codes in Tables E1 and E2 in this article’s Online Repository at www.jaci-global.org). The diagnosis date of asthma and allergy was the date when the child fulfilled both criteria for asthma and allergy. Follow-up started 1 year after the diagnosis, and concluded at age 19 years, emigration, death, a change in exposure (eg, if a dog died or was acquired), or study end (December 31, 2023), whichever occurred first. Both a depiction of the study design (Fig 1) and a flowchart of the study population (Fig E1 in this article’s Online Repository at www.jaci-global.org) are provided. The Regional Ethical Review Board in Stockholm (Dnr 2018/1697-31/1; 2023-03916-02) approved the study and waived the requirement for informed consent.

### Variables

In this study population of children with established asthma and allergy, the outcome variables were

• Moderate-to-severe asthma (yes/no) at 2-, 4-, and 6-year follow-up was assessed based on asthma medications dispensed throughout the respective follow-up year, using adapted Global Initiative for Asthma treatment steps.^2^ Children were classified to the “yes” category if, during that year, they received >1 controller medication (such as a combination of inhaled corticosteroids [ICS] and leukotriene receptor antagonists, or ICS and long-acting β-2 agonists, or if they were dispensed long-acting β-2 agonists or biologics). The “no” category included children who, during the year, received ICS monotherapy, leukotriene receptor antagonist monotherapy, short-acting β-2 agonists (SABAs), or no asthma medications. Detailed medicine codes are provided in Table E1.
• Asthma exacerbation was assessed throughout the follow-up time based on events of asthma emergency visits (defined as emergency department visit or unplanned specialist visit due to asthma) and the high use of SABA, separately. Recurrent emergency visits within 2 weeks were counted as 1 event. The number of dispensed SABAs was measured in each follow-up year and categorized as “high SABA use” if there were ≥5 dispensed prescriptions within a year.

Household exposure to dogs, defined as a parent registered as a dog owner, was the main exposure. All dogs residing in Sweden must be ID-marked and registered to an owner with the Swedish Agriculture Board.^20^ The registration of ownership should be completed before the dog reaches age 4 months, or within 4 weeks if there is an ownership change.^20^ Moreover, ∼70% of all dogs, mainly purebreds, are also registered by the Swedish Kennel Club.^21^ Household exposure to dogs was categorized as

• No dog exposure: Neither parent registered as a dog owner at the time of diagnosis or any point during follow-up.
• Continuous dog exposure: At least 1 parent registered as dog owner at diagnosis and throughout the follow-up.
• Discontinued dog exposure:
  ○ For moderate-to-severe asthma outcome: At least 1 parent registered as the dog owner at diagnosis, and discontinuation of the ownership occurred before the year the outcome was assessed.
  ○ For asthma exacerbation outcome: At least 1 parent registered as the dog owner at diagnosis, and discontinuation of the ownership before follow-up started.

Discontinuation of the parental dog ownership was considered if the dog was registered to a new owner or if the dog died. In cases where the dog's death was unknown, ownership was assumed to end after the dog had reached 10 years of age.^22^, ^23^, ^24^

Other variables were included as covariates. Initial asthma severity was determined by dispensed asthma medications at baseline.^2^ Parental asthma was defined as asthma in a biological parent, identified using the same algorithm as for the study population.^17^ Population density (/km^2^) was based on the municipality where the child resided at baseline. Parental education was categorized by the highest attained education at baseline. Parental country of birth was categorized as “Nordic,” :”Europe except Nordic,” and “others.” Detailed definitions of each covariate appear in Table E3 in this article’s Online Repository (available at www.jaci-global.org). These covariates were chosen *a priori* based on a directed acyclic graph (see Fig E2 in this article’s Online Repository at www.jaci-global.org).

### Data sources

A data linkage of various registers was made by Statistics Sweden and the National Board of Health and Welfare using Sweden’s unique personal identity number. The study population was identified from the linkage between the Total Population Register and the Medical Birth Register. Dog owners were identified from the National Dog Register held by the Swedish Board of Agriculture and the Dog Register held by the Swedish Kennel Club. Asthma and allergy diagnoses from inpatient and outpatient specialized care were ascertained from the National Patient Register. Dispensed asthma medications were identified through the Prescribed Drug Register. Living area was obtained from the Total Population Register. Parental education was provided by the longitudinal integrated database for health insurance and labor market studies. Further details about each register in Table E4 in this article’s Online Repository (available at www.jaci-global.org).

### Statistical analysis

Descriptive statistics are presented as proportions for categorical variables and medians (quartile 1 [Q1], quartile 3 [Q3]) for continuous variables. Baseline characteristics in each exposure group are presented. To assess the association between exposure to dogs and moderate-to-severe asthma at 2-, 4-, and 6-year follow-up points, logistic regression was performed. Unadjusted odds ratio (OR), adjusted OR (controlling for all the covariates including baseline asthma severity), and their 95% CIs were estimated. These estimates were obtained for the entire study population and separately for subgroups based on age of diagnosis onset (ie, ages <6 years and ≥6 years).

To assess the association between exposure to dogs and the risk of exacerbations assessed as emergency asthma visits, a Cox-based shared-frailty model was used.^25^ This approach was chosen to handle recurrent event data by incorporating a random effect to account for within-individual correlation. Independence between event and censoring times was assumed. The time since the start of follow-up (1 year after diagnosis) was used as the analysis time scale. The incidence rate of emergency asthma visits by exposure group was calculated. Unadjusted and adjusted hazard ratios were estimated.

To assess the association between exposure to dogs and the risk of exacerbations assessed as high SABA use, the yearly repeated measurements throughout follow-up years were applied to generalized estimating equations for longitudinal data, specifying a binomial family, logit link, and unstructured covariance matrix to account for within-individual correlation. Unadjusted and adjusted ORs and their 95% CIs were estimated.

Some sensitivity analyses were performed including: excluding children with life-limiting conditions (eg, neurodegenerative disorders, inherited muscular dystrophy);^26^ modifying the lifespan of the dogs to 8 or 12 years in those dogs with unknown death dates; excluding children whose household dog exposure ended before their asthma diagnosis; restricting the analysis to the subpopulation of first-born children; restricting the analysis to the subpopulation of children with separated parents; stratifying the analysis before and after 2016 to account for improvements in the coverage of the dog register over time; and stratifying the analysis by geographical area. Analysis was performed using SAS version 9.4 (SAS Institute, Cary, NC) and Stata version 18 (StataCorp, College Station, Tex). No *a priori* sample size calculation was performed because the study used national register data.

## Results

### Study population

Of the 99,389 children with both asthma and airway allergy, 13,980 (14.1%) had household exposure to dogs at the time of diagnosis. Of those with exposure to dogs, 12,759 (91.3%) had continuous exposure during the study period. Compared to the nonexposed, the continuous dog exposure group had a higher proportion of moderate-severe asthma at baseline (16.3% vs 14.9%) but a lower proportion of emergency asthma visits (5.6% vs 6.6%). The baseline characteristics between the discontinued dog exposure and the continuous dog exposure groups were similar. Compared to the nonexposed group, household dog-exposed children had a higher proportion of parents born in Nordic countries and lower parental university education (see Table I).

**Table I:** Characteristics of the study population

	All	No exposure	Discontinued dog exposure	Continuous dog exposure
	(n = 99,389)	(n = 85,409)	(n = 1,221)	(n = 12,759)
Sex				
Male	58,614 (59.0)	50,922 (59.6)	691 (56.6)	7,001 (54.9)
Female	40,775 (41.0)	34,487 (40.4)	530 (43.4)	5,758 (45.1)
Age (y)	6.6 (4.6, 9.8)	6.5 (4.5, 9.6)	6.4 (4.6, 9.5)	7.4 (4.8, 11.2)
Asthma severity at baseline∗				
Mild	84,343 (84.9)	72,653 (85.1)	1,014 (83.1)	10,676 (83.7)
Moderate-severe	15,046 (15.1)	12,756 (14.9)	207 (16.9)	2,083 (16.3)
Emergency asthma visit at baseline∗				
No	92,985 (93.6)	79,787 (93.4)	1,157 (94.8)	12,041 (94.4)
Yes	6,404 (6.4)	5,622 (6.6)	64 (5.2)	718 (5.6)
High SABA use at baseline∗				
No	95,579 (96.2)	82,159 (96.2)	1,181 (96.7)	12,239 (95.9)
Yes	3,810 (3.8)	3,250 (3.8)	40 (3.3)	520 (4.1)
Population density (/km^2^)	468 (187, 1231)	451 (187, 1178)	783 (344, 1489)	699 (328, 1428)
Parental asthma				
No	55,814 (56.2)	48,154 (56.4)	695 (56.9)	6,965 (54.6)
Yes	43,575 (43.8)	37,255 (43.6)	526 (43.1)	5,794 (45.4)
Parents' highest attained education				
Primary school or below	2,892 (2.9)	2,384 (2.8)	53 (4.3)	455 (3.6)
High school	35,093 (35.3)	28,134 (32.9)	599 (49.1)	6,360 (49.9)
University degree or higher	61,404 (61.8)	54,891 (64.3)	569 (46.6)	5,944 (46.6)
Father's country of birth				
Nordic	81,320 (81.8)	68,448 (80.1)	1,137 (93.1)	11,735 (92.0)
Europe except Nordic	5,647 (5.7)	5,180 (6.1)	25 (2.9)	432 (3.4)
Other countries	12,422 (12.5)	11,781 (13.8)	49 (4.0)	592 (4.6)
Mother's country of birth				
Nordic	82,207 (82.7)	69,195 (81.0)	1,140 (93.4)	11,872 (93.1)
Europe except Nordic	5,055 (5.1)	4,651 (5.4)	34 (2.8)	370 (2.9)
Other countries	12,127 (12.2)	11,563 (11.6)	47 (3.8)	517 (4.0)

Categorical variables are presented as n (%). Continuous variables are presented as median (Q1, Q3).

∗ Baseline period was measured from time of diagnosis to 364 days after diagnosis.

### The associations between exposure to dog and moderate-to-severe asthma at 2, 4, and 6 years of follow-up

Children who remained at 2-, 4-, and 6-year follow-up had similar baseline characteristics to the overall population (see Fig E3 in this article’s Online Repository at www.jaci-global.org). Proportion of dog exposure by regions are shown in Fig E4 in this article’s Online Repository (available at www.jaci-global.org). At 2-year follow-up, the prevalence of moderate-to-severe asthma was 15.0% (nonexposed), 16.4% (discontinued dog exposure), and 16.1% (continuous dog exposure). The continuous dog exposure group had slightly higher odds of moderate-to-severe asthma at the 2-year follow-up in the unadjusted analysis (unadjusted OR: 1.09; 95% CI: 1.03-1.16; *P* = .005), but no longer significant after adjustment (adjusted OR: 1.00; 95% CI: 0.94-1.07; *P* = .326) (see Table II). No significant association was observed between discontinued dog exposure and moderate-to-severe asthma at the 2-year follow-up in either the unadjusted or adjusted analysis (adjusted OR: 1.07; 95% CI: 0.93-1.23; *P* = .948). Similarly, at the 4- and 6-year follow-up, no significant associations between exposure groups and moderate-to-severe asthma were observed (Table II). Results of regression models showing sequential adjustment for covariates, from sex and age to the fully adjusted model, are presented in Table E5 in this article’s Online Repository (available at www.jaci-global.org).

**Table II:** Association between exposure to dogs and moderate-to-severe asthma at 2-, 4-, and 6-year follow-ups

Exposure groups	2-y follow-up			4-y follow-up			6-y follow-up		
	n/ N (%)	Unadjusted OR (95% CI)	Adjusted OR (95% CI)	n/ N	Unadjusted OR (95% CI)	Adjusted OR (95% CI)	n/ N	Unadjusted OR (95% CI)	Adjusted OR (95% CI)
Total population									
No dog exposure	10,810/ 72,280 (15.0)	Ref	Ref	8,299/ 53,798 (15.4)	Ref	Ref	6,363/ 38,152 (16.7)	Ref	Ref
Discontinued dog exposure	301/ 1,839 (16.4)	1.11 (0.98-1.26)	1.07 (0.93-1.23)	424/ 2,551 (16.6)	1.09 (0.98-1.22)	1.05 (0.94-1.18)	409/ 2,365 (17.3)	1.04 (0.94-1.17)	1.01 (0.90-1.13)
Continuous dog exposure	1,423/ 8,841 (16.1)	1.09 (1.03-1.16)∗∗	1.00 (0.94-1.07)	846/ 5,137 (16.5)	1.08 (1.00-1.17)∗	1.03 (0.95-1.12)	448/ 2,799 (16.0)	0.95 (0.86-1.06)	0.92 (0.82-1.03)
Age 3-6 y									
No dog exposure	5,450/ 41,042 (13.3)	Ref	Ref	4,732/ 33,208 (14.2)	Ref	Ref	4,237/ 26,042 (16.3)	Ref	Ref
Discontinued dog exposure	187/ 1,115 (16.8)	1.32 (1.12-1.54)∗∗	1.26 (1.05-1.51)∗	270/ 1,693 (15.9)	1.14 (0.99-1.31)	1.10 (0.96-1.27)	294/ 1,670 (17.6)	1.10 (0.97-1.25)	1.06 (0.93-1.21)
Continuous dog exposure	512/ 4,047 (12.7)	0.95 (0.86-1.04)	0.91 (0.81-1.02)	385/ 2,576 (14.9)	1.06 (0.94-1.18)	1.04 (0.92-1.17)	261/ 1,638 (15.9)	0.98 (0.85-1.12)	0.95 (0.82-1.09)
Age 7-16 y									
No dog exposure	55,360/ 31,238 (17.2)	Ref	Ref	3,567/ 20,590 (17.3)	Ref	Ref	2,126/ 12,110 (17.6)	Ref	Ref
Discontinued dog exposure	114/724 (15.8)	0.90 (0.74-1.10)	0.86 (0.69-1.08)	154/858 (17.9)	1.04 (0.87-1.25)	1.00 (0.83-1.21)	115/695 (16.6)	0.93 (0.76-1.14)	0.89 (0.72-1.10)
Continuous dog exposure	911/4,794 (19.0)	1.13 (1.05-1.22)∗∗	1.07 (0.98-1.17)	461/2,561 (18.0)	1.05 (0.94-1.17)	1.01 (0.90-1.13)	187/1,161 (16.1)	0.90 (0.77-1.07)	0.88 (0.74-1.04)

ORs were estimated using logistic regression. The adjusted models included sex, age, asthma severity at baseline, population density, parental asthma, parental education, and parental country of birth.

*n/N*, number of individuals with moderate-to-severe asthma/total number of individuals in the respective groups.

∗*P* < .05; ∗∗*P* < .01.

When examining changes in asthma severity from baseline to the 2-year follow-up, the proportion of children whose asthma transitioned from mild to moderate-to-severe asthma was 7.3% in the nonexposed group, 8.3% in the discontinued dog exposure group, and 7.6% in the continuous dog exposure group. Conversely, the proportion of children whose asthma improved from moderate-to-severe to no longer having moderate-to-severe asthma was 6.7% in the nonexposed group, 7.4% in the discontinued dog exposure group, and 7.3% in the continuous dog exposure group. The changes in asthma severity from baseline to the 2-, 4-, and 6-year follow-up by exposure group are presented in Fig E5.

### The associations between exposure to dogs and asthma exacerbations during pediatric years

During follow-up (median: 6.0 [Q1, Q3: 2.8, 8.6] years), a total of 8651 events of emergency asthma visits were observed in 5989 children. Of all the children with events, 1701 (28.4%) experienced recurrent events. The lowest incidence rate of emergency asthma visits was found in the nonexposed (14.6 per 1000 person-years) group, followed by the continuous dog exposure group (15.1 per 1000 person-years), and the discontinued dog exposure group (22.1 per 1000 person-years). The association between continuous dog exposure compared to nonexposed and the risk of emergency asthma visits was apparent after adjustment for the covariates (adjusted hazard ratio: 1.17; 95% CI: 1.06-1.20; *P* = .002) (see Table III).

**Table III:** Association between exposure to dogs and emergency asthma visits during follow-up

	No. events	No. children with the event	Incidence rate∗ (95% CI)	Unadjusted		Adjusted	
				HR (95% CI)	*P*	HR (95% CI)	*P*
No dog exposure	7729	5172	14.6 (14.3-14.9)	Ref	Ref	Ref	Ref
Discontinued dog exposure	108	67	22.1 (18.3-26.7)	1.41 (1.06-1.87)	.017	1.52 (1.15-2.00)	.003
Continuous dog exposure	814	750	15.1 (14.1-16.2)	1.02 (0.93-1.13)	.678	1.17 (1.06-1.29)	.002

HRs and their 95% CIs were estimated using Cox-based shared-frailty model allowing for repeated events of emergency asthma visits. Age at baseline, sex, initial asthma severity, population density, parental asthma, parental education, and parental country of birth were included in the adjusted model.

*HR*, Hazard ratio.

∗ Incidence rates are presented per 1000 person-years.

Of the study population, 5120 children had high SABA use during follow-up. At the 2-year follow-up, the proportion of children with high SABA use was 1.8% in the nonexposed, 2.8% in the discontinued dog exposure, and 1.7% in the continuous dog exposure groups. At the 4-year follow-up, the corresponding proportions were 1.5%, 1.7%, and 2.0%, respectively. Results from longitudinal generalized estimating equation analysis showed that, compared to the nonexposed, continuous dog exposure was associated with 1.2 times higher odds of high SABA use (adjusted OR: 1.25; 95% CI: 1.12-1.39; *P* < .001) over the follow-up period. Both unadjusted and adjusted analyses showed a similar magnitude of association (see Table IV).

**Table IV:** Association between exposure to dogs and high SABA use throughout follow-up years

	No. children with high SABA use	Unadjusted		Adjusted	
		OR (95% CI)	*P*	OR (95% CI)	*P*
No dog exposure	4563	Ref	Ref	Ref	Ref
Discontinued dog exposure	51	1.16 (0.85-1.60)	.348	1.12 (0.82-1.53)	.484
Continuous dog exposure	506	1.23 (1.11-1.36)	<.001	1.27 (1.13-1.41)	<.001

ORs and 95% CIs were estimated using generalized estimating equations for longitudinal data, applying binomial family, logit link, and unstructured covariance. The adjusted models included sex, age, asthma severity at baseline, population density, parental asthma, parental education, and parental country of birth.

### Sensitivity analysis

The null associations between continuous dog exposure and moderate-to-severe asthma measured at the 2-, 4-, and 6-year follow-ups remained across various sensitivity analyses (see Table E6-E14 in this article’s Online Repository at www.jaci-global.org). Similarly, the associations observed between continuous dog exposure and a slightly increased risk of emergency asthma visits or high SABA use also persisted with modifying the dog lifespan assumption (see Tables E15 and E16 in this article’s Online Repository at www.jaci-global.org).

## Discussion

In this nationwide cohort of children with asthma and airway allergy, no association between continuous household exposure to dogs and moderate-to-severe asthma at 2-, 4-, and 6-year follow-up was observed. Yet, children with continuous exposure to dogs had a slightly higher risk of asthma exacerbation, indicated by emergency visits and SABA use, than the nonexposed children. Interestingly, asthma outcomes between the continuous dog exposure and discontinued dog exposure groups did not differ.

Studies assessing the long-term effects of household exposure to dogs in children with asthma and allergy are scarce. In a Korean cohort of children with atopic asthma, pet ownership was not associated with asthma severity or fractional exhaled nitric oxide level after 12 months of follow-up.^16^ Cross-sectional studies have reported mixed findings. A large American study in individuals found both sensitization and exposure to dogs were associated with increased risk of asthma attacks in individuals ≥6 years.^12^ Conversely, 2 other American studies found no association between dog exposure and life-threatening asthma in children.^14^^,^^15^ A Finnish study of 206 children with asthma reported fewer symptoms and lower blood eosinophilia among those exposed to dogs.^13^ These studies were limited by single time-point exposure or outcome measures or potential recall bias. Different measures of asthma outcomes may also contribute to the various findings. In contrast, our study used multiple registers with high coverage, non–self-reported measurements, and the ability to follow both exposure and outcome over time. Furthermore, it assessed both asthma severity and exacerbation risk, providing a more comprehensive understanding of the impact of continuous and discontinued household exposure to dogs on different dimensions of asthma outcomes.

Consistent with a previous study in children with atopic allergy followed for 1 year,^16^ our study did not demonstrate that continuous household exposure to dogs was associated with risk of moderate-to-severe asthma at 2, 4, and 6 years of follow-up, independently of baseline asthma severity, sex, age, and sociodemographic factors. Additionally, changes in asthma severity from baseline to follow-up were similar across exposure groups, suggesting that dog exposure does not affect severity over time. Because severity is commonly assessed by treatment level,^2^ our findings indicate that, in children with allergic asthma, continuous exposure to dogs does not affect the level of treatment required to control asthma symptoms over the long term. Nonetheless, potential biases (such as variation in treatment adherence and differences in physicians’ management practices) should be considered when interpreting these findings.

Different findings emerged when assessing asthma exacerbations. Continuous exposure to dogs was associated with an increased risk of high SABA use and emergency visits due to asthma. Similarly, previous studies demonstrated association between dog exposure and asthma attack in children and adults with allergic asthma^12^ and life-threatening asthma in children.^14^^,^^15^ Although pet ownership may affect indoor endotoxin, the association between dog exposure and asthma exacerbation appears independent of endotoxin levels.^12^ While sensitization to major dog allergens from dander, saliva, urine, and glands (such as Can f 1–Can f 6) is known to be associated with the risk of uncontrolled asthma,^11^^,^^27^ our study adds that continuous exposure to dogs in children with allergic asthma may slightly increase the risk of exacerbation in a median follow-up of 6 years. Nevertheless, as long-term pet exposure may induce tolerance,^28^ this association could vary by exposure duration. Whether dog exposure affects the severity of the exacerbation episodes warrants further investigation.

Although closely related, asthma severity and exacerbation are not interchangeable. A child can have rather mild asthma and experience exacerbation and vice versa. Our findings on the association between dog exposure and different asthma outcomes suggest that continuous exposure to dogs does not appear to affect asthma treatment but may provoke acute exacerbations in children with allergic asthma. Future studies with clinical measures (such as asthma control test, peak expiratory flow, spirometry) measured longitudinally would be valuable to confirm and further explore these findings. While dog exposure in the household contributes to increased levels of dog allergens in the home environment, it may also contribute to other factors (such as increased physical activity and altered microbiota),^29^^,^^30^ which could help explain the neutral effect on asthma severity alongside the elevated risk of exacerbation. Alternatively, the null association with severity alongside the increased risk of exacerbations observed in children with continuous dog exposure may suggest suboptimal treatment in this group.

An important and practical question is often raised by patients: Will it be better for my child’s asthma if we discontinue dog ownership? The present study indicated that overall asthma outcomes did not differ between the group with continuous and discontinued household dog exposure. This finding may be partly explained by the persistence of the dog exposure; it takes at least several months for pet allergen levels to drop significantly after the pet is removed,^31^ and pet allergens are not completely absent even in pet-free households.^32^ Moreover, pet allergens are almost unavoidable in public areas such as schools, where the allergen concentrations often exceed those in pet-free households.^33^^,^^34^ It is also important to note that the discontinued dog exposure group in the present study did not exclusively represent families who actively relinquish the dog, as the reason for discontinued exposure (eg, natural death or other circumstances) was unknown. While these findings were at the group level, at an individual level factors such as having a high degree of polysensitization^11^ and patients’ emotional attachment to their dogs^35^ should ultimately be considered in clinical decision-making. Additionally, while it is reasonable to assume that baseline asthma severity might influence a family's decision to own and keep dogs, our study found only a modest difference in baseline asthma severity between the exposure groups, suggesting this factor may not have been a primary driver of dog ownership decisions in our cohort.

To our knowledge, this was the first nationwide longitudinal study able to estimate household exposure to dogs and assess its association with recurrent events of asthma exacerbations throughout pediatric years using data sources with high validity and coverage. Nevertheless, some limitations should be acknowledged. First, despite the high accuracy of diagnosis of asthma and allergy in the study population,^17^ data on dog allergen sensitization and specific asthma phenotypes were not available. However, prior studies from South Korea and the United States found that the association between dog exposure and pediatric asthma outcomes did not differ by dog-specific sensitization.^16^^,^^36^ Therefore, the lack of sensitization data in this study is unlikely to have altered the observed association. Second, parental dog ownership may not fully capture the duration and intensity of the exposure. Moreover, for children with divorced parents, accurately tracking continuous household dog exposure becomes challenging, increasing the potential for misclassification bias. Third, information of dog’s death date was incomplete and dog lifespan of 10 years was assumed. While misclassification bias of exposure is probable, multiple sensitivity analyses were performed (eg, by modifying the assumption of the dog’s lifespan and analyzing the subpopulation with separated parents), and the results remain consistent. Additionally, the information on other pet exposure was unavailable. Moreover, information on whether dogs were kept indoors or outdoors was not available in this study. Nevertheless, most dogs in Sweden typically reside indoors with their owners. Only certain breeds are permitted by regulation to live outdoors year-round.^37^ Lastly, the generalizability of these findings to other populations may be affected by variations in cultural practices related to dog ownership, the prevalence of dog ownership, outdoor environmental factors (eg, climate), and indoor environmental factors (eg, wall and floor materials^38^).

### Conclusion

In children with asthma and airway allergy, continuous household exposure to dogs does not seem to be associated with increased risk of moderate-to-severe asthma. However, it is associated with slightly increased risk of asthma exacerbations. The asthma outcomes do not differ between children with continuous and discontinued dog exposure. These findings suggest that keeping dogs in households with children who have asthma and allergy may not affect overall asthma severity but could trigger exacerbations.

**Key messages**

• In a population of children with asthma and allergy followed over 6 years, those who had continuous dog exposure showed no increased risk of moderate-to-severe asthma but had a 1.2-fold higher risk of asthma exacerbation compared to the nonexposed children.
• Long-term asthma outcomes did not differ between the group who had continuous dog exposure and the group who had discontinued exposure after diagnosis of asthma and allergy.
• Understanding the magnitude of the effect of continuous and discontinued dog exposure on asthma outcomes can help clinicians provide more nuanced, evidence-based counselling regarding environmental control strategies.

## Disclosure statement

This work was supported by the 10.13039/501100004359Swedish Research Council (grant 2023-02327), the 10.13039/501100003793Swedish Heart-Lung Foundation (grant 20240974), the Swedish Asthma and Allergy Association Research Fund (grant 2024-0010), as well as grants provided by Region Stockholm (ALF project RS2022-0674), the Strategic Research Program in Epidemiology at Karolinska Institutet, and the Foundation Frimurare Barnhuset in Stockholm. The funder had no role in the design, data collection, data analysis, and reporting of this study.

Disclosure of potential conflict of interest: The authors declare that they have no relevant conflicts of interest.
