# Supplementary material for: Dog exposure and subsequent asthma outcomes in children with asthma and allergy
Source: J Allergy Clin Immunol Glob. 2026 Mar 20;5(3):100692. doi: 10.1016/j.jacig.2026.100692 (PMC13091991; doi:10.1016/j.jacig.2026.100692)
Supplement: Supplementary Figs and Tables [file mmc1.docx]

Supplemental file

Association between continuous dog exposure and asthma outcomes in children with asthma and allergy

Resthie R Putri, Cecilia Lundholm, Bronwyn K Brew, Hanna Karim, Jon R Konradsen, Tove Fall, Catarina Almqvist

Contents

[Table E1. Asthma ascertainment based on ICD-10 codes and/ or ATC codes 2](#_Toc226017974)

[Table E2. Allergy ascertainment based on ICD-10 codes and/ or ATC codes 3](#_Toc226017975)

[Figure E1. Flowchart of the study population 4](#_Toc226017976)

[Table E3. Definition of the covariates 5](#_Toc226017977)

[Figure E2. Directed acyclic graph of the association between dog exposure and asthma outcomes in a population of children with asthma and allergy 6](#_Toc226017978)

[Table E4. Data sources 7](#_Toc226017979)

[Figure E3. Baseline characteristics of children in the analysis of 2-, 4-, and 6-year follow-up for the outcome of an asthma visit or medication 8](#_Toc226017980)

[Figure E4. Proportion of household exposure to dogs among children with asthma and allergy by region 9](#_Toc226017981)

[Table E5. Association between dog exposure and moderate-to-severe asthma with sequential adjustments for covariates 10](#_Toc226017982)

[Figure E5. Change in asthma severity from baseline to follow-up by dog exposure groups 11](#_Toc226017983)

[Table E6. Association between dog exposure and moderate-to-severe asthma after excluding children with life-limiting conditions 13](#_Toc226017984)

[Table E7. Association between dog exposure and moderate-to-severe asthma, assuming dog’s lifespan of 8 years in those with missing dog’s death data 14](#_Toc226017985)

[Table E8. Association between dog exposure and moderate-to-severe asthma, assuming dog’s lifespan of 12 years and 12 years in those with missing dog’s death data 15](#_Toc226017986)

[Table E9. Association between dog exposure and moderate-to-severe asthma after excluding children whose household dog exposure ended before their asthma diagnosis 16](#_Toc226017987)

[Table E10. Association between dog exposure and moderate-to-severe-asthma in the subpopulation of first-born children 17](#_Toc226017988)

[Table E11. Association between dog exposure and moderate-to-severe asthma in the subpopulation of children whose parents lived separately 18](#_Toc226017989)

[Table E12. Association between dog exposure and moderate-to-severe asthma in children who were diagnosed with asthma and allergy before year 2016 19](#_Toc226017990)

[Table E13. Association between dog exposure and moderate-to-severe asthma in children who were diagnosed with asthma and allergy from year 2016 onwards 20](#_Toc226017991)

[Table E14. Association between dog exposure and moderate-to-severe asthma in children who were diagnosed with asthma and allergy by geographical area 21](#_Toc226017992)

[Table E15. Hazard ratios of the association between dog exposure and the risk of emergency asthma visit, assuming dog’s lifespan of 8 years and 12 years in those with missing dog’s death data 22](#_Toc226017993)

[Table E16. Odds ratios of the association between dog exposure and high use SABA, assuming dog’s lifespan of 8 years and 12 years in those with missing dog’s death data 23](#_Toc226017994)

# Table E1. Asthma ascertainment based on ICD-10 codes and/ or ATC codes

| **ICD-10 codes (obtained from the National Patient Register)**  J45 Asthma  J46 Status asthmaticus |
| --- |
| **ATC codes (obtained from the Prescribed Drug Register)**  R03BA Glucocorticoids, inhalants  R03AC Selective beta-2-adrenoreceptor agonists, inhalants  R03AK Adrenergics in combination with corticosteroids or other drugs, excl. anticholinergics, inhalants  R03DC Leukotriene receptor antagonists |
| Asthma diagnosis was ascertained using a validated algorithm by Örtqvist, 2013.   - Briefly, for individuals aged 4.5 years or above, an individual has to have (a) at least a diagnosis of asthma based on ICD-10 codes, *or* (b) at least 2 dispensed of inhaled glucocorticoids , combination inhalants, or leukotriene receptor antagonists, *or* (c) at least 3 dispenses of inhaled beta-2-adrenoreceptor agonists, inhaled glucocorticoids, combination inhalants, or leukotriene receptor antagonists within a year. - For individuals aged below 4.5 years, to ascertain asthma diagnosis, an individual has to fulfill criterion (a) above and either criterion (b) or criterion (c). |

The positive predictive value of the asthma diagnosis by these criteria in children aged >4.5 to 17 years was 94%.

Reference:

Örtqvist, A.K., Lundholm, C., Wettermark, B., Ludvigsson, J.F., Ye, W. and Almqvist, C. (2013), Validation of asthma and eczema in population-based Swedish drug and patient registers. Pharmacoepidemiol Drug Saf, 22: 850-860. https://doi.org/10.1002/pds.3465

# Table E2. Allergy ascertainment based on ICD-10 codes and/ or ATC codes

Rhinoconjunctivitis was ascertained using the previous algorithm (1) by fulfilling 1 of the 4 criteria below.

| **Criterion 1**  At least 1 ICD-10 codes:  J30 Allergic rhinitis and vasomotor rhinitis  J31.0 Chronic rhinitis |
| --- |
| **Criterion 2**  At least 2 dispensed prescriptions of ATC codes:  R01AD01 – R01AD60 nasal preparation of corticosteroid  *And*  Without any of the ICD-10 codes below:  J33 Nasal polyps  J01 Acute sinusitis  J32 Chronic sinusitis |
| **Criterion 3**  At least 2 dispensed prescriptions of ATC codes:  R06A Antihistamines for systemic use  And  Without any of the ICD-10 codes below:  L29 Pruritus  DL50 Allergic urticaria |
| **Criterion 4**  At least 1 dispensed prescription of ATC code:  V01A Specific immune therapy, allergen subtract therapy  S01GX Medication for allergic conjunct |

Using disease- specific hospital contacts and/or filled prescriptions of disease- specific medication, the sensitivity was 84% and specificity was 82% for capturing children with allergic rhinoconjunctivitis (2).

Reference:

1. Henriksen L, Simonsen J, Haerskjold A, et al. Incidence rates of atopic dermatitis, asthma, and allergic rhinoconjunctivitis in Danish and Swedish children. J Allergy Clin Immunol. 2015;136(2):360-6.e2. doi:10.1016/j.jaci.2015.02.003
2. Stensballe LG, Klansø L, Jensen A, Hærskjold A, Thomsen SF, Simonsen J. The validity of register data to identify children with atopic dermatitis, asthma or allergic rhinoconjunctivitis. Pediatric Allergy and Immunology. 2017;28(6):535-542. doi:10.1111/pai.12743

# Figure E1. Flowchart of the study population


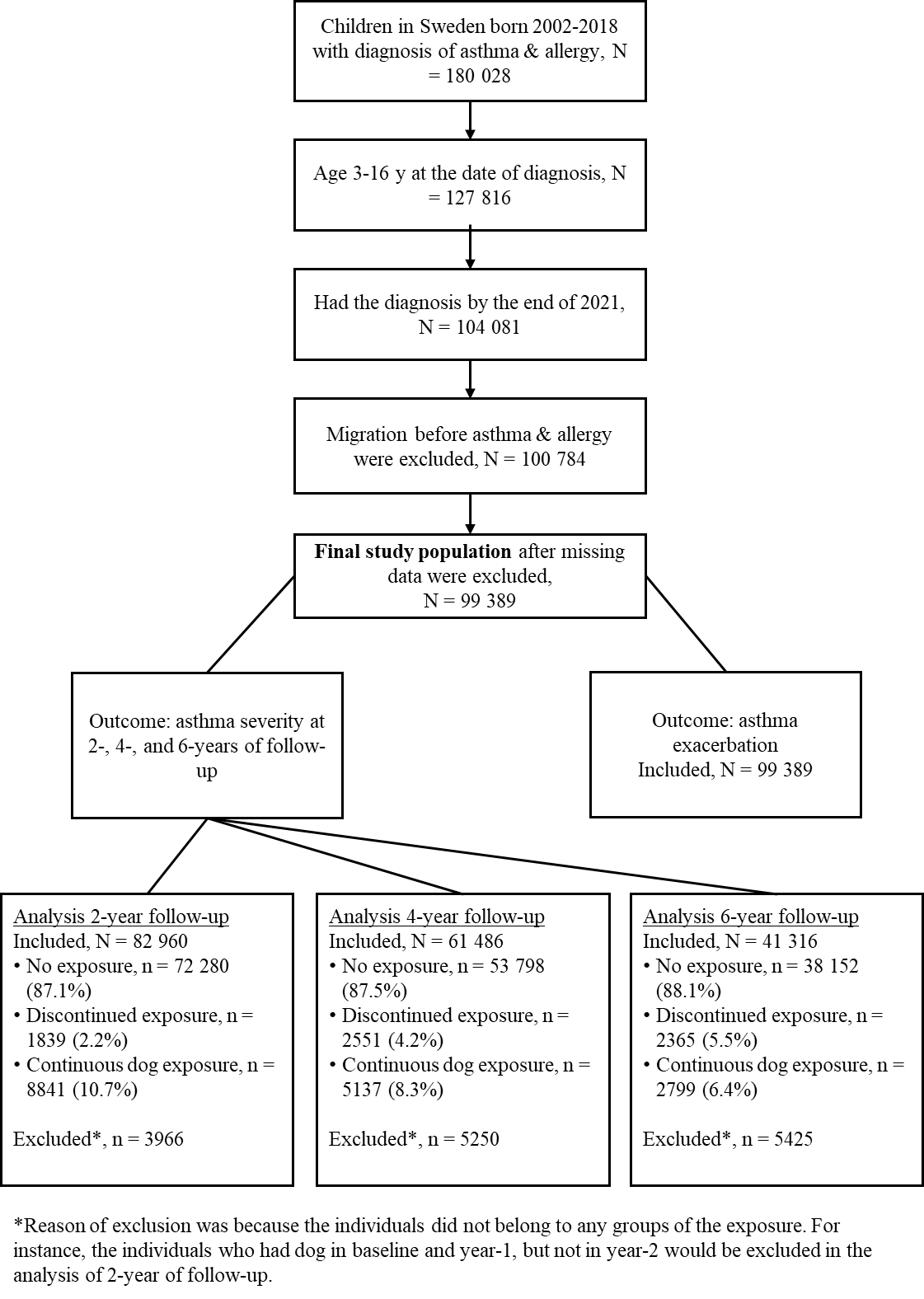


# Table E3. Definition of the covariates

| **Variable** | **Data source** | **Definition** |
| --- | --- | --- |
| Initial asthma severity | Prescribed Drug Register | It was defined based on dispensed asthma medications within a year after diagnosis, and divided into two categories.   - ‘Mild asthma’: if the study participant was prescribed only inhaled corticosteroid (ICS), or only leukotriene receptor antagonist (LRTA), or short-acting β2 agonist (SABA). - ‘Moderate-severe asthma’: if the study participant was prescribed long-acting β2 agonist (LABA), combination therapy (e.g., ICS + LABA, ICS + LRTA, ICS + LABA + LRTA), or biologics. |
| Parental asthma | Patient Register and Prescribed Drug Register | Identified using the same algorithm as in the study population, see Supplemental Table 1. This was a dichotomous variable. ‘Yes’ if at least one of the parents had diagnosis of asthma. |
| Population density | Total Population Register | The municipalities where the study participants resided at the time of asthma and allergy diagnosis were identified. Data on population density in each municipality for the respective years were obtained from Statistics Sweden (https://www.scb.se/hitta-statistik/sverige-i-siffror/manniskorna-i-sverige/befolkningstathet-i-sverige/). Population density was treated as a continuous variable (number of inhabitants per square kilometer). |
| Parental education | The longitudinal integrated database for health insurance and labour market studies | Was based on the highest attained education from both parents at the year when the child’s asthma and allergy was diagnosed. This variable was divided into: ‘primary school or below’ (9 years of education or less), ‘high school’ (10-12 years), and ‘university degree or higher (more than 12 years). |
| Parental country of birth | Total Population Register | Was divided into three categories:   - ‘Nordic’ if the parent was born in Sweden, Denmark, Norway, Finland or Iceland. - ‘Europe except Nordic’ if the parent was born in a European country but non-Nordic countries. - ‘Others’ if the parent was born outside Europe. |

# Figure E2. Directed acyclic graph of the association between dog exposure and asthma outcomes in a population of children with asthma and allergy


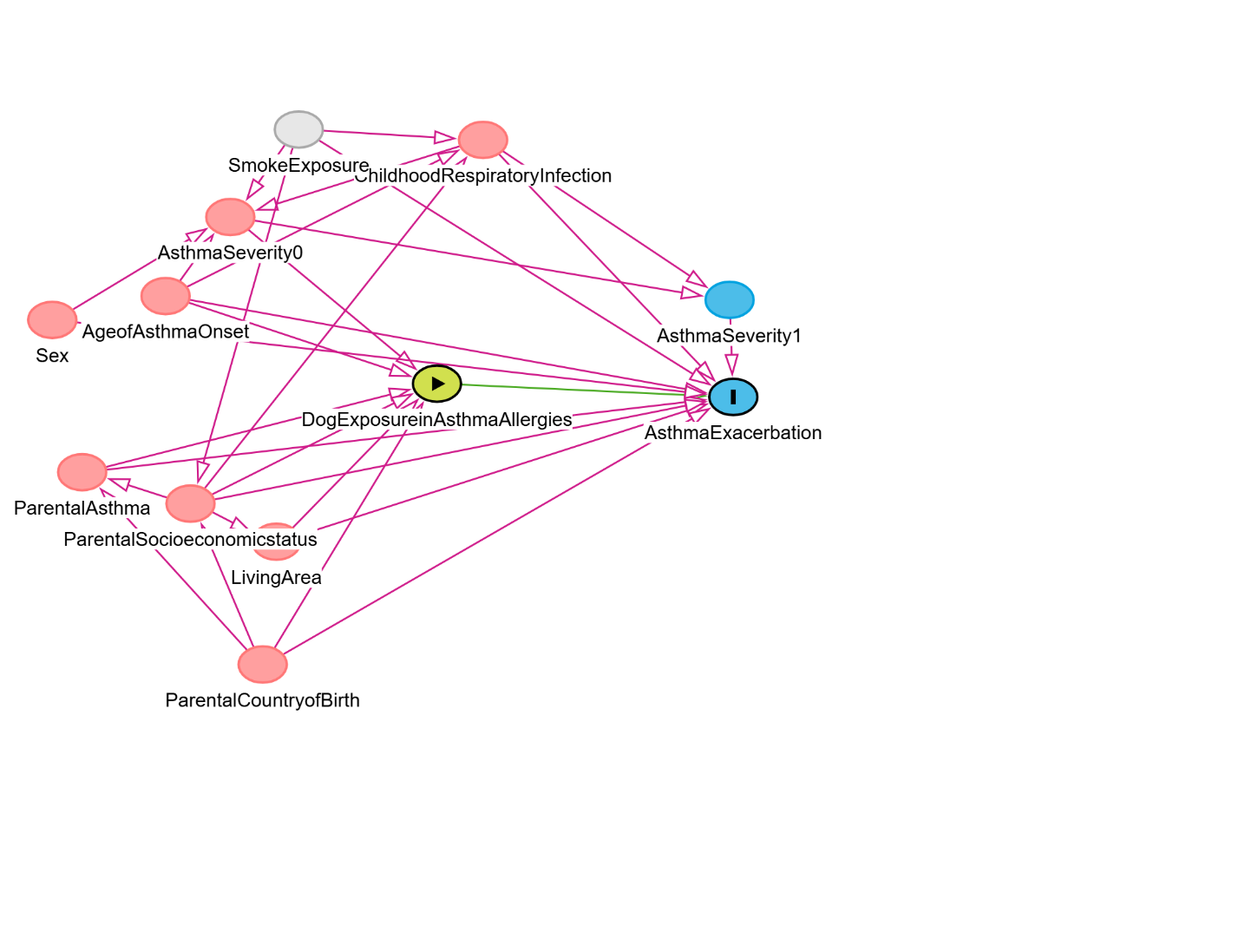


Note: while it has been known that exposure to dogs in early life is associated with a lower risk of asthma, the present study focused on dog exposure in a population with established asthma and allergy. Hence, the early life exposure was not included in the directed acyclic graph.

# Table E4. Data sources

| **Register’s name** | **Description** |
| --- | --- |
| Total Population Register | Together with the Medical Birth Register, this register was used in the present study to identify the study population, as well as their biological parents. It was also utilized to obtain data on living area, migration and the parents’ country of birth. The register started in 1968 and covers all individuals living in Sweden (1). |
| Medical Birth Register | This register started in 1973, and it records births of the Swedish residents born in Sweden. The coverage is 97-99% (2). |
| Dog register from the Swedish Board of Agriculture | The register started in 2001 and intended to cover all dogs residing in Sweden. Each dog must be registered with its owner. The mandatory registration of dogs has been applied by law since 2001. The e-service for registration of dog exposure started in 2013 (3). |
| Dog register from the Swedish Kennel Club | The register started in 1976 for pedigree dogs, show dogs, and breeding purpose. Registration of dogs in this register is voluntary. Breeders and owners who are members of the club can register their dogs (4). |
| National Patient Register | This register records data on diagnoses and procedures in inpatient care (became national since 1987) and outpatient specialized care (since 2001). Given that all specialized health care is obliged to report to the register, the coverage of the register is estimated to be high. In general, the diagnoses recorded in the register have high positive predictive value (5). |
| Prescribed Drug Register | This register was used in the present study to identify dispensed asthma medication. This register was established in July 2004 and contains all prescribed medications dispensed in pharmacies (6). |
| The cause of death register | This was used to retrieve information of death and death date in the present study. The register started in 1961 and has high coverage (7). |
| The longitudinal integrated database for health insurance and labour market studies (LISA) | This database was used in the present study to obtain data of the parents’ highest attained education. This database covers the adults (aged 16 years or above) residing in Sweden. This database started in 1990 and collects data on education, income, and occupation annually (8). |

References:

1. Ludvigsson JF, Almqvist C, Bonamy AK, et al. Registers of the Swedish total population and their use in medical research. Eur J Epidemiol. 2016;31(2):125-136. doi:10.1007/s10654-016-0117-y
2. Cnattingius S, Källén K, Sandström A, et al. The Swedish medical birth register during five decades: documentation of the content and quality of the register. Eur J Epidemiol. 2023;38(1):109-120. doi:10.1007/s10654-022-00947-5
3. The Swedish Board of Agriculture. Identification and registration of dogs. <https://jordbruksverket.se/languages/english/swedish-board-of-agriculture/animals/identification-and-registration-of-animals/identification-and-registration-of-dogs>.
4. Svenska Kennelklubben. Hundregister (in Swedish). <https://hundar.skk.se/hunddata/About.aspx?Avdelning=HUND>.
5. Everhov ÅH, Frisell T, Osooli M, et al. Diagnostic accuracy in the Swedish national patient register: a review including diagnoses in the outpatient register. Eur J Epidemiol. Published online March 27, 2025. doi:10.1007/s10654-025-01221-0
6. Wettermark B, Hammar N, Fored CM, et al. The new Swedish Prescribed Drug Register--opportunities for pharmacoepidemiological research and experience from the first six months. Pharmacoepidemiol Drug Saf. 2007;16(7):726-735. doi:10.1002/pds.1294
7. Brooke HL, Talbäck M, Hörnblad J, et al. The Swedish cause of death register. Eur J Epidemiol. 2017;32(9):765-773. doi:10.1007/s10654-017-0316-1
8. Ludvigsson JF, Svedberg P, Olén O, Bruze G, Neovius M. The longitudinal integrated database for health insurance and labour market studies (LISA) and its use in medical research. Eur J Epidemiol. 2019;34(4):423-437. doi:10.1007/s10654-019-00511-8

# Figure E3. Baseline characteristics of children in the analysis of 2-, 4-, and 6-year follow-up for the outcome of an asthma visit or medication


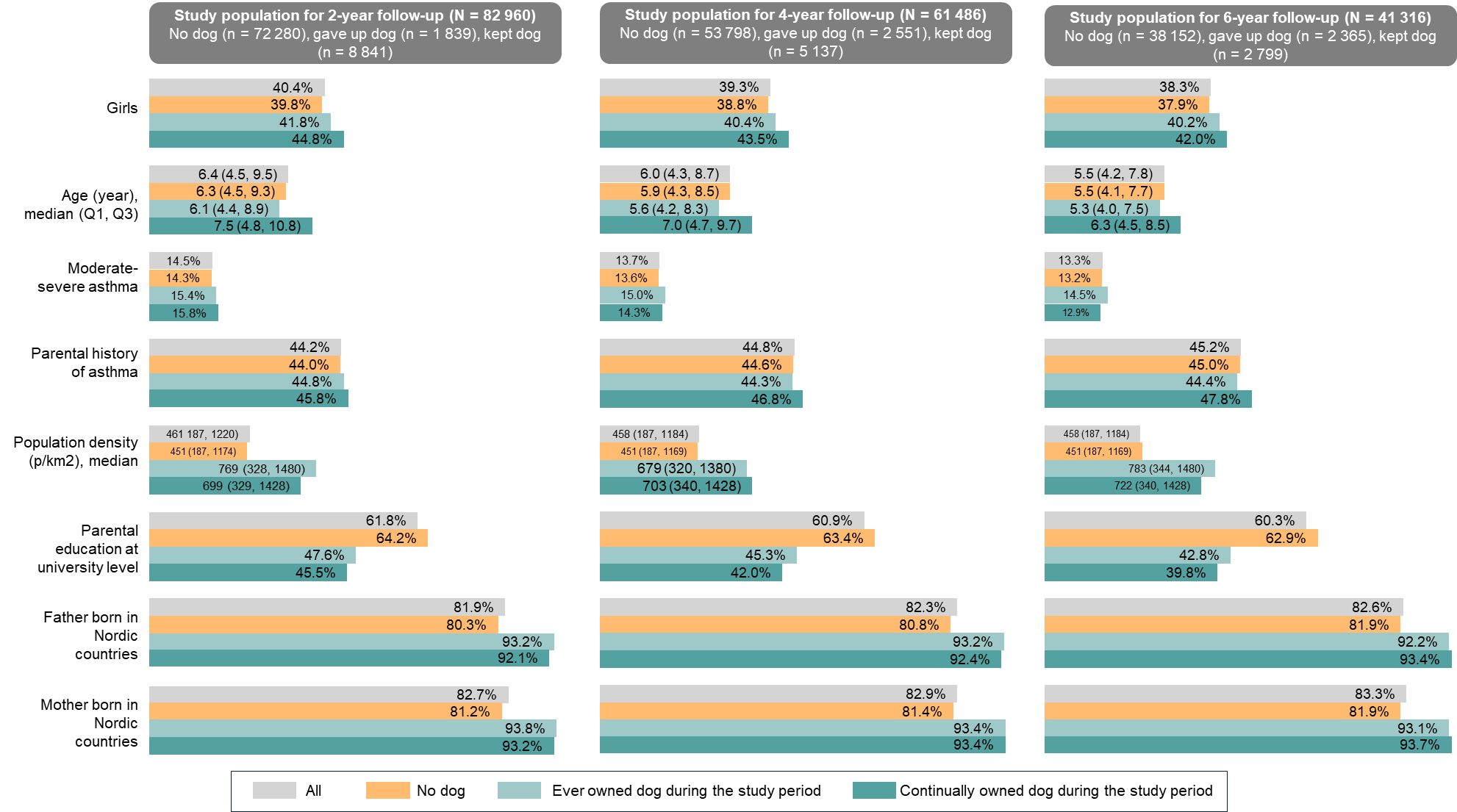


# Figure E4. Proportion of household exposure to dogs among children with asthma and allergy by region


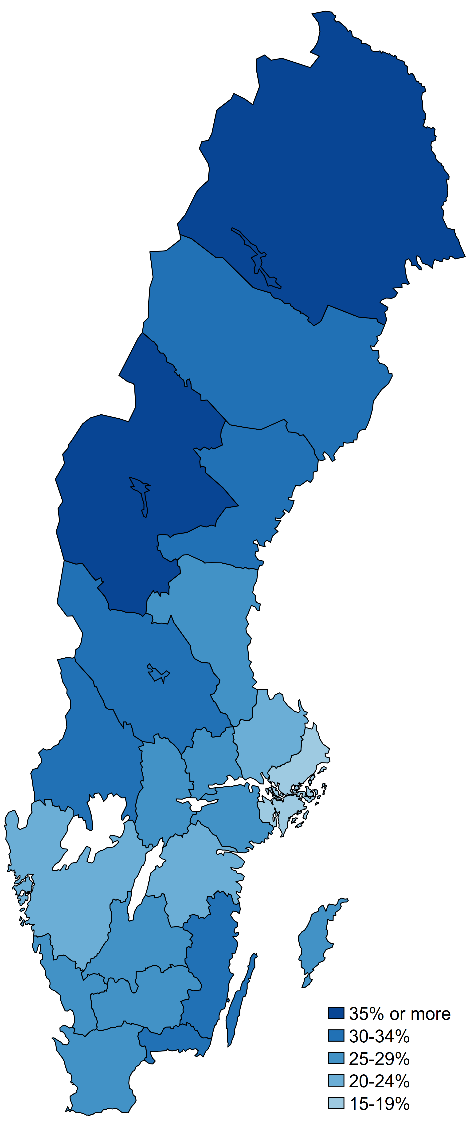


# Table E5. Association between dog exposure and moderate-to-severe asthma with sequential adjustments for covariates

| Exposure groups | OR (95% CI) | | | |
| --- | --- | --- | --- | --- |
|  | Model 1 | Model 2 | Model 3 | Model 4 |
|  |  |  |  |  |
| 2-year follow-up | | | | |
| No dog exposure | Ref | Ref | Ref | Ref |
| Discontinued dog exposure | 1.11 (0.98–1.26) | 1.12 (0.99-1.27) | 1.09 (0.96-1.24) | 1.07 (0.93–1.23) |
| Continuous dog exposure | 1.09 (1.03–1.16)** | 1.04 (0.98-1.11) | 1.01 (0.95-1.08) | 1.00 (0.94–1.07) |
|  |  |  |  |  |
| 4-year follow-up | | | | |
| No dog exposure | Ref | Ref | Ref | Ref |
| Discontinued dog exposure | 1.09 (0.98–1.22) | 1.09 (0.98-1.22) | 1.08 (0.97-1.20) | 1.05 (0.94–1.18) |
| Continuous dog exposure | 1.08 (1.00–1.17)* | 1.05 (0.98-1.14) | 1.02 (0.94-1.11) | 1.03 (0.95–1.12) |
|  |  |  |  |  |
| 6-year follow-up | | | | |
| No dog exposure | Ref | Ref | Ref | Ref |
| Discontinued dog exposure | 1.04 (0.94–1.17) | 1.05 (0.94-1.17) | 1.02 (0.91-1.14) | 1.01 (0.90–1.13) |
| Continuous dog exposure | 0.95 (0.86–1.06) | 0.94 (0.85-1.05) | 0.91 (0.82-1.01) | 0.92 (0.82–1.03) |
|  |  |  |  |  |

* P <0.05; ** P <0.01

Model 1: Unadjusted.

Model 2: Adjusted for sex and age.

Model 3: Adjusted for sex, age, residential density, parental asthma, parental socioeconomic status, parental country of birth.

Model 4: Adjusted for sex, age, residential density, parental asthma, parental socioeconomic status, parental country of birth, baseline asthma severity.

# Figure E5. Change in asthma severity from baseline to follow-up by dog exposure groups


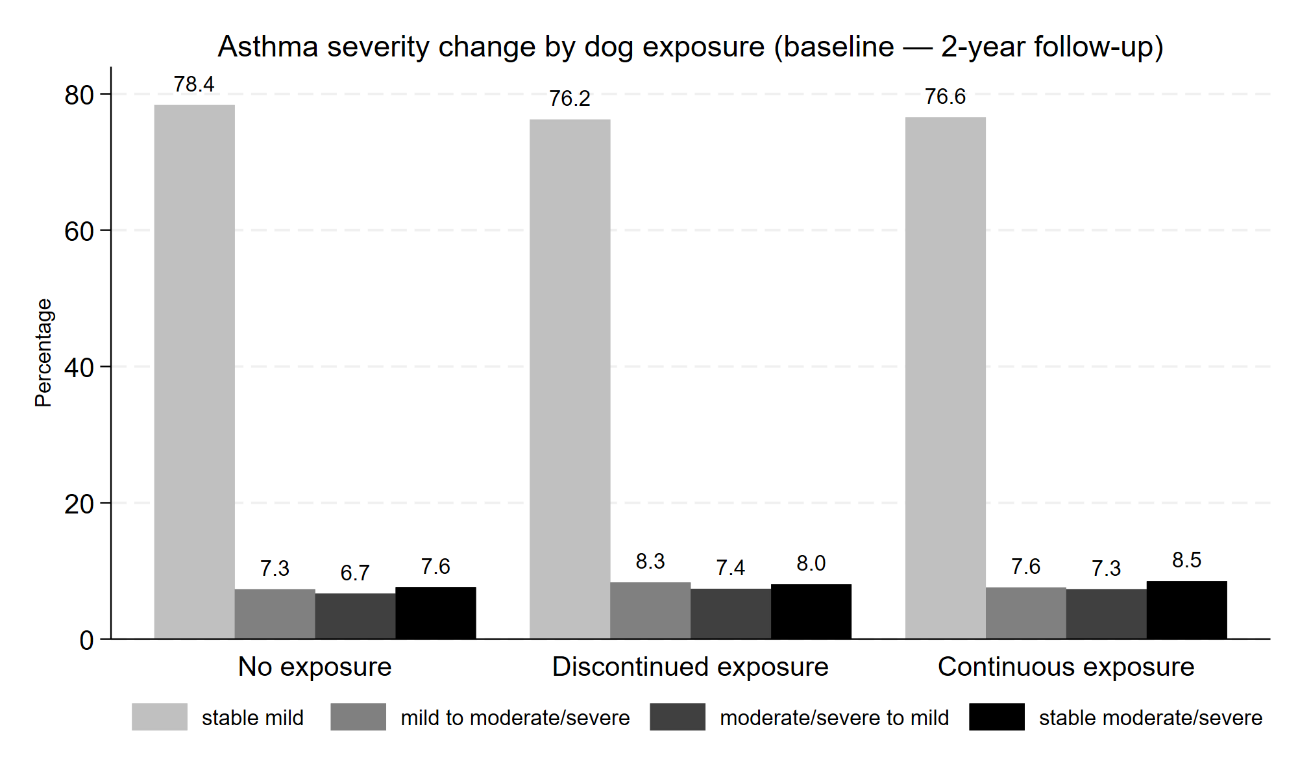


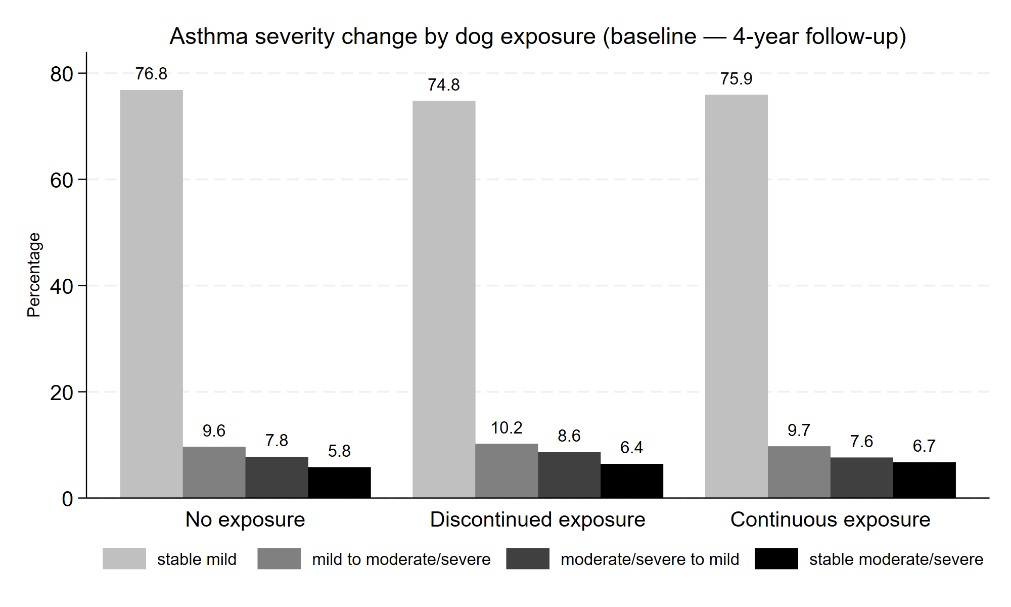


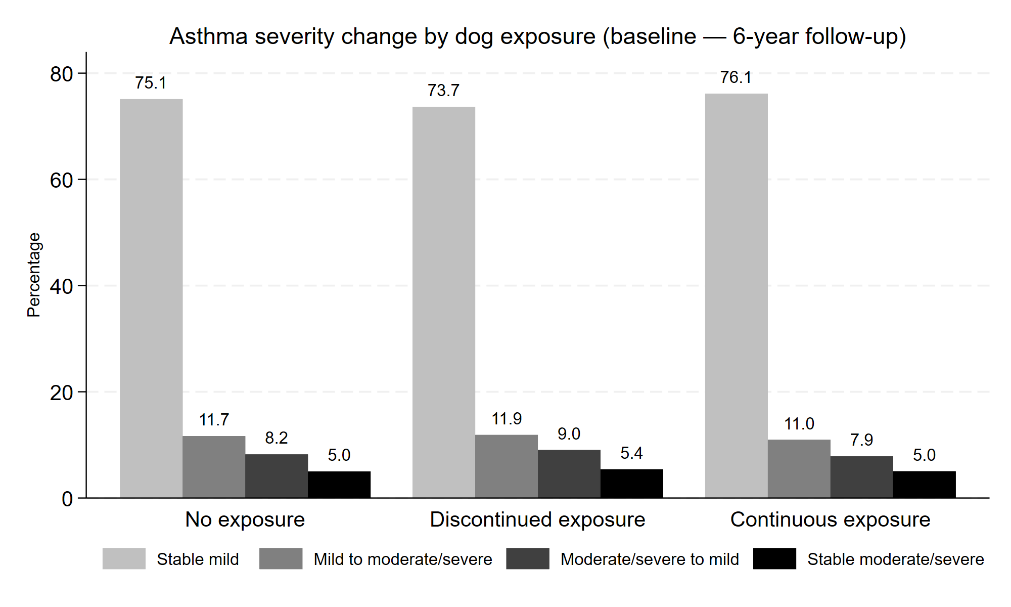


# Table E6. Association between dog exposure and moderate-to-severe asthma after excluding children with life-limiting conditions

| Exposure groups | 2-year follow-up | | 4-year follow-up | | 6-year follow-up | |
| --- | --- | --- | --- | --- | --- | --- |
|  | Unadjusted OR (95% CI) | Adjusted OR (95% CI) | Unadjusted OR (95% CI) | Adjusted OR (95% CI) | Unadjusted OR (95% CI) | Adjusted OR (95% CI) |
| **All** | | | | | | |
| No dog exposure | Ref | Ref | Ref | Ref | Ref | Ref |
| Discontinued dog exposure | 1.11 (0.98-1.26) | 1.07 (0.93-1.23) | 1.08 (0.97-1.20) | 1.04 (0.93-1.17) | 1.03 (0.92-1.15) | 0.99 (0.89-1.12) |
| Continuous dog exposure | 1.09 (1.03-1.16)** | 1.00 (0.94-1.08) | 1.08 (1.00-1.17)* | 1.03 (0.95-1.12) | 0.96 (0.86-1.06) | 0.93 (0.83-1.03) |
|  |  |  |  |  |  |  |
| **Age 3-6 years at baseline** | | | | | | |
| No dog exposure | Ref | Ref | Ref | Ref | Ref | Ref |
| Discontinued dog exposure | 1.31 (1.11-1.54)** | 1.25 (1.03-1.49)* | 1.11 (0.98-1.28) | 1.08 (0.94-1.25) | 1.09 (0.95-1.24) | 1.05 (0.92-1.21) |
| Continuous dog exposure | 0.94 (0.85-1.04) | 0.91 (0.82-1.02) | 1.06 (0.94-1.19) | 1.05 (0.93-1.19) | 0.97 (0.85-1.12) | 0.95 (0.82-1.09) |
|  |  |  |  |  |  |  |
| **Age 7-16 years at baseline** | | | | | | |
| No dog exposure | Ref | Ref | Ref | Ref | Ref | Ref |
| Discontinued dog exposure | 0.91 (0.74-1.11) | 0.87 (0.69-1.09) | 1.04 (0.87-1.24) | 1.00 (0.83-1.21) | 0.92 (0.74-1.13) | 0.88 (0.71-1.09) |
| Continuous dog exposure | 1.13 (1.05-1.22)** | 1.07 (0.97-1.17) | 1.05 (0.94-1.17) | 1.01 (0.90-1.13) | 0.91 (0.77-1.08) | 0.89 (0.75-1.05) |

* P <0.05; ** P <0.01

# Table E7. Association between dog exposure and moderate-to-severe asthma, assuming dog’s lifespan of 8 years in those with missing dog’s death data

| Exposure groups | 2-year follow-up | | 4-year follow-up | | 6-year follow-up | |
| --- | --- | --- | --- | --- | --- | --- |
|  | Unadjusted OR (95% CI) | Adjusted OR (95% CI) | Unadjusted OR (95% CI) | Adjusted OR (95% CI) | Unadjusted OR (95% CI) | Adjusted OR (95% CI) |
| **All** | | | | | | |
| No dog exposure | Ref | Ref | Ref | Ref | Ref | Ref |
| Discontinued dog exposure | 1.02 (0.90-1.15) | 0.89 (0.78-1.02) | 1.11 (1.00-1.23) | 1.05 (0.94-1.17) | 0.98 (0.89-1.10) | 0.94 (0.84-1.05) |
| Continuous dog exposure | 1.09 (1.02-1.17)* | 1.01 (0.94-1.09) | 1.05 (0.96-1.15) | 1.00 (0.92-1.11) | 0.98 (0.86-1.12) | 0.95 (0.83-1.09) |
|  |  |  |  |  |  |  |
| **Age 3-6 years at baseline** | | | | | | |
| No dog exposure | Ref | Ref | Ref | Ref | Ref | Ref |
| Discontinued dog exposure | 1.03 (0.87-1.21) | 0.90 (0.75-1.09) | 1.12 (0.98-1.29) | 1.10 (0.95-1.27) | 1.03 (0.90-1.18) | 1.00 (0.88-1.15) |
| Continuous dog exposure | 0.92 (0.82-1.03) | 0.88 (0.77-1.00)* | 1.03 (0.91-1.17) | 1.02 (0.89-1.17) | 0.93 (0.79-1.11) | 0.89 (0.75-1.08) |
|  |  |  |  |  |  |  |
| **Age 7-16 years at baseline** | | | | | | |
| No dog exposure | Ref | Ref | Ref | Ref | Ref | Ref |
| Discontinued dog exposure | 1.02 (0.86-1.21) | 0.88 (0.72-1.07) | 1.08 (0.92-1.26) | 1.03 (0.87-1.21) | 0.90 (0.75-1.08) | 0.86 (0.71-1.04) |
| Continuous dog exposure | 1.14 (1.04-1.24)** | 1.09 (0.99-1.20) | 1.03 (0.91-1.16) | 0.99 (0.86-1.13) | 1.03 (0.84-1.27) | 1.02 (0.82-1.26) |

* P <0.05; ** P <0.01

# Table E8. Association between dog exposure and moderate-to-severe asthma, assuming dog’s lifespan of 12 years and 12 years in those with missing dog’s death data

| Exposure groups | 2-year follow-up | | 4-year follow-up | | 6-year follow-up | |
| --- | --- | --- | --- | --- | --- | --- |
|  | Unadjusted OR (95% CI) | Adjusted OR (95% CI) | Unadjusted OR (95% CI) | Adjusted OR (95% CI) | Unadjusted OR (95% CI) | Adjusted OR (95% CI) |
| **All** | | | | | | |
| No dog exposure | Ref | Ref | Ref | Ref | Ref | Ref |
| Discontinued dog exposure | 1.05 (0.91-1.21) | 1.02 (0.87-1.19) | 1.11 (0.99-1.25) | 1.11 (0.98-1.25) | 1.07 (0.96-1.21) | 1.03 (0.92-1.16) |
| Continuous dog exposure | 1.09 (1.03-1.15)** | 1.00 (0.94-1.06) | 1.07 (0.99-1.13) | 1.01 (0.94-1.09) | 0.95 (0.87-1.04) | 0.92 (0.83-1.00) |
|  |  |  |  |  |  |  |
| **Age 3-6 years at baseline** | | | | | | |
| No dog exposure | Ref | Ref | Ref | Ref | Ref | Ref |
| Discontinued dog exposure | 1.17 (0.97-1.42) | 1.18 (0.95-1.46) | 1.23 (1.06-1.42)** | 1.22 (1.05-1.42)* | 1.15 (1.01-1.32)* | 1.11 (0.96-1.28) |
| Continuous dog exposure | 1.00 (0.92-1.09) | 0.95 (0.86-1.05) | 1.05 (0.95-1.16) | 1.04 (0.94-1.16) | 0.97 (0.86-1.09) | 0.94 (0.83-1.07) |
|  |  |  |  |  |  |  |
| **Age 7-16 years at baseline** | | | | | | |
| No dog exposure | Ref | Ref | Ref | Ref | Ref | Ref |
| Discontinued dog exposure | 0.93 (0.75-1.15 | 0.86 (0.67-1.08) | 0.96 (0.79-1.17) | 0.93 (0.75-1.15) | 0.90 (0.72-1.14) | 0.85 (0.67-1.08) |
| Continuous dog exposure | 1.11 (1.03-1.19)** | 1.03 (0.95-1.13) | 1.03 (0.93-1.13) | 0.98 (0.88-1.09) | 0.91 (0.79-1.05) | 0.87 (0.75-1.01) |

* P <0.05; ** P <0.01

# Table E9. Association between dog exposure and moderate-to-severe asthma after excluding children whose household dog exposure ended before their asthma diagnosis

| Exposure groups | 2-year follow-up | | 4-year follow-up | | 6-year follow-up | |
| --- | --- | --- | --- | --- | --- | --- |
|  | Unadjusted OR (95% CI) | Adjusted OR (95% CI) | Unadjusted OR (95% CI) | Adjusted OR (95% CI) | Unadjusted OR (95% CI) | Adjusted OR (95% CI) |
| **All** | | | | | | |
| No dog exposure | Ref | Ref | Ref | Ref | Ref | Ref |
| Discontinued dog exposure | 1.12 (0.99-1.27) | 1.08 (0.94-1.24) | 1.10 (0.99-1.22) | 1.06 (0.95-1.18) | 1.05 (0.94-1.17) | 1.01 (0.90-1.13) |
| Continuous dog exposure | 1.10 (1.04-1.17)** | 1.01 (0.94-1.08) | 1.09 (1.00-1.17) | 1.03 (0.95-1.12) | 0.95 (0.86-1.06) | 0.92 (0.82-1.02) |
|  |  |  |  |  |  |  |
| **Age 3-6 years at baseline** | | | | | | |
| No dog exposure | Ref | Ref | Ref | Ref | Ref | Ref |
| Discontinued dog exposure | 1.33 (1.13-1.56)** | 1.27 (1.06-1.52)* | 1.15 (1.00-1.31) | 1.11 (0.96-1.28) | 1.10 (0.97-1.26) | 1.06 (0.93-1.22) |
| Continuous dog exposure | 0.95 (0.86-1.05) | 0.92 (0.82-1.02) | 1.06 (0.95-1.19) | 1.05 (0.93-1.18) | 0.98 (0.85-1.12) | 0.95 (0.82-1.09) |
|  |  |  |  |  |  |  |
| **Age 7-16 years at baseline** | | | | | | |
| No dog exposure | Ref | Ref | Ref | Ref | Ref | Ref |
| Discontinued dog exposure | 0.91 (0.74-1.11) | 0.87 (0.69-1.08) | 1.05 (0.88-1.25) | 1.00 (0.83-1.21) | 0.94 (0.76-1.15) | 0.89 (0.72-1.11) |
| Continuous dog exposure | 1.14 (1.06-1.23)** | 1.07 (0.98-1.17) | 1.05 (0.94-1.17) | 1.00 (0.89-1.12) | 0.90 (0.77-1.06) | 0.88 (0.74-1.04) |

* P <0.05; ** P <0.01

# Table E10. Association between dog exposure and moderate-to-severe-asthma in the subpopulation of first-born children

| Exposure groups | 2-year follow-up | | 4-year follow-up | | 6-year follow-up | |
| --- | --- | --- | --- | --- | --- | --- |
|  | Unadjusted OR (95% CI) | Adjusted OR (95% CI) | Unadjusted OR (95% CI) | Adjusted OR (95% CI) | Unadjusted OR (95% CI) | Adjusted OR (95% CI) |
| **All** | | | | | | |
| No dog exposure | Ref | Ref | Ref | Ref | Ref | Ref |
| Discontinued dog exposure | 1.08 (0.90-1.29) | 1.05 (0.86-1.28) | 1.05 (0.90-1.22) | 1.00 (0.85-1.17) | 1.01 (0.87-1.19) | 0.98 (0.84-1.16) |
| Continuous dog exposure | 1.07 (0.97-1.17) | 0.98 (0.89-1.09) | 1.04 (0.92-1.17) | 0.98 (0.85-1.11) | 0.98 (0.83-1.16) | 0.97 (0.81-1.15) |
|  |  |  |  |  |  |  |
| **Age 3-6 years at baseline** | | | | | | |
| No dog exposure | Ref | Ref | Ref | Ref | Ref | Ref |
| Discontinued dog exposure | 1.21 (0.96-1.52) | 1.09 (0.85-1.41) | 1.00 (0.83-1.21) | 0.92 (0.76-1.13) | 1.00 (0.83-1.19) | 0.95 (0.79-1.15) |
| Continuous dog exposure | 0.94 (0.82-1.09) | 0.89 (0.75-1.05) | 1.03 (0.87-1.24) | 1.00 (0.83-1.21) | 0.95 (0.76-1.19) | 0.94 (0.74-1.18) |
|  |  |  |  |  |  |  |
| **Age 7-16 years at baseline** | | | | | | |
| No dog exposure | Ref | Ref | Ref | Ref | Ref | Ref |
| Discontinued dog exposure | 0.96 (0.72-1.29) | 0.95 (0.68-1.32) | 1.24 (0.96-1.61) | 1.15 (0.88-1.52) | 1.10 (0.81-1.51) | 1.03 (0.74-1.43) |
| Continuous dog exposure | 1.12 (0.99-1.27) | 1.07 (0.93-1.23) | 1.00 (0.85-1.17) | 0.96 (0.80-1.15) | 0.99 (0.77-1.28) | 1.02 (0.78-1.33) |

* P <0.05; ** P <0.01

# Table E11. Association between dog exposure and moderate-to-severe asthma in the subpopulation of children whose parents lived separately

| Exposure groups | 2-year follow-up | | 4-year follow-up | | 6-year follow-up | |
| --- | --- | --- | --- | --- | --- | --- |
|  | Unadjusted OR (95% CI) | Adjusted OR (95% CI) | Unadjusted OR (95% CI) | Adjusted OR (95% CI) | Unadjusted OR (95% CI) | Adjusted OR (95% CI) |
| **All** | | | | | | |
| No dog exposure | Ref | Ref | Ref | Ref | Ref | Ref |
| Discontinued dog exposure | 1.02 (0.80-1.31) | 1.05 (0.80-1.38) | 0.98 (0.80-1.20) | 0.97 (0.78-1.19) | 1.00 (0.82-1.22) | 1.01 (0.82-1.24) |
| Continuous dog exposure | 1.03 (0.92-1.16) | 0.99 (0.87-1.13) | 1.07 (0.93-1.24) | 1.05 (0.91-1.22) | 0.86 (0.71-1.04) | 0.86 (0.70-1.05) |
|  |  |  |  |  |  |  |

* P <0.05; ** P <0.01

Stratified analysis by age group at baseline was not performed due to limited sample size.

# Table E12. Association between dog exposure and moderate-to-severe asthma in children who were diagnosed with asthma and allergy before year 2016

| Exposure groups | 2-year follow-up | | 4-year follow-up | | 6-year follow-up | |
| --- | --- | --- | --- | --- | --- | --- |
|  | Unadjusted OR (95% CI) | Adjusted OR (95% CI) | Unadjusted OR (95% CI) | Adjusted OR (95% CI) | Unadjusted OR (95% CI) | Adjusted OR (95% CI) |
| **All** | | | | | | |
| No dog exposure | Ref | Ref | Ref | Ref | Ref | Ref |
| Discontinued dog exposure | 1.11 (0.91-1.36) | 1.05 (0.84-1.32) | 1.14 (0.99-1.31) | 1.09 (0.94-1.26) | 1.06 (0.94-1.20) | 1.02 (0.90-1.16) |
| Continuous dog exposure | 1.01 (0.92-1.11) | 0.95 (0.85-1.06) | 1.05 (0.95-1.16) | 0.99 (0.89-1.10) | 0.93 (0.83-1.04) | 0.90 (0.80-1.01) |
|  |  |  |  |  |  |  |

* P <0.05; ** P <0.01

Stratified analysis by age group at baseline was not performed due to limited sample size.

# Table E13. Association between dog exposure and moderate-to-severe asthma in children who were diagnosed with asthma and allergy from year 2016 onwards

| Exposure groups | 2-year follow-up | | 4-year follow-up | | 6-year follow-up | |
| --- | --- | --- | --- | --- | --- | --- |
|  | Unadjusted OR (95% CI) | Adjusted OR (95% CI) | Unadjusted OR (95% CI) | Adjusted OR (95% CI) | Unadjusted OR (95% CI) | Adjusted OR (95% CI) |
| **All** | | | | | | |
| No dog exposure | Ref | Ref | Ref | Ref | Ref | Ref |
| Discontinued dog exposure | 1.09 (0.93-1.28) | 1.07 (0.89-1.28) | 1.02 (0.86-1.20) | 1.00 (0.84-1.19) | 0.96 (0.74-1.25) | 0.92 (0.70-1.21) |
| Continuous dog exposure | 1.14 (1.06-1.24)** | 1.04 (0.95-1.14) | 1.13 (1.00-1.28)* | 1.09 (0.96-1.24) | 1.09 (0.85-1.41) | 1.03 (0.79-1.34) |
|  |  |  |  |  |  |  |

* P <0.05; ** P <0.01

Stratified analysis by age group at baseline was not performed due to limited sample size.

# Table E14. Association between dog exposure and moderate-to-severe asthma in children who were diagnosed with asthma and allergy by geographical area

| Exposure groups | 2-year follow-up | | 4-year follow-up | | 6-year follow-up | |
| --- | --- | --- | --- | --- | --- | --- |
|  | Unadjusted OR (95% CI) | Adjusted OR (95% CI) | Unadjusted OR (95% CI) | Adjusted OR (95% CI) | Unadjusted OR (95% CI) | Adjusted OR (95% CI) |
| **Northern Sweden** | | | | | | |
| No dog exposure | Ref | Ref | Ref | Ref | Ref | Ref |
| Discontinued dog exposure | 1.15 (0.86-1.55) | 1.09 (0.78-1.54) | 1.01 (0.77-1.33) | 1.00 (0.75-1.34) | 1.10 (0.85-1.43) | 1.07 (0.82-1.40) |
| Continuous dog exposure | 1.09 (0.94-1.26) | 0.98 (0.83-1.16) | 1.05 (0.87-1.27) | 0.97 (0.79-1.19) | 1.00 (0.78-1.28) | 0.96 (0.74-1.24) |
|  |  |  |  |  |  |  |
| **Central Sweden** | | | | | | |
| No dog exposure | Ref | Ref | Ref | Ref | Ref | Ref |
| Discontinued dog exposure | 0.92 (0.73-1.17) | 0.91 (0.70-1.17) | 1.09 (0.91-1.32) | 1.05 (0.86-1.27) | 1.03 (0.85-1.24) | 1.00 (0.82-1.21) |
| Continuous dog exposure | 1.04 (0.94-1.16) | 0.96 (0.85-1.07) | 1.07 (0.93-1.22) | 1.03 (0.89-1.19) | 0.95 (0.79-1.14) | 1.03 (0.79-1.34) |
|  |  |  |  |  |  |  |
| **Southern Sweden** | | | | | | |
| No dog exposure | Ref | Ref | Ref | Ref | Ref | Ref |
| Discontinued dog exposure | 1.15 (0.98-1.38) | 1.12 (0.93-1.38) | 1.07 (0.92-1.24) | 1.04 (0.89-1.22) | 0.99 (0.84-1.16) | 0.92 (0.70-1.21) |
| Continuous dog exposure | 1.07 (0.98-1.16) | 1.02 (0.93-1.13) | 1.04 (0.93-1.17) | 1.02 (0.91-1.15) | 0.89 (0.77-1.04) | 1.03 (0.79-1.34) |

# Table E15. Hazard ratios of the association between dog exposure and the risk of emergency asthma visit, assuming dog’s lifespan of 8 years and 12 years in those with missing dog’s death data

|  | Unadjusted HR (95% CI) | Adjusted HR (95% CI) |
| --- | --- | --- |
|  |  |  |
| *Assuming dog’s lifespan of 8 years in those with missing dog’s death data* | | |
| No dog exposure | Ref | Ref |
| Discontinued dog exposure | 0.90 (0.67 - 1.20) | 0.88 (0.65 - 1.18) |
| Continuous dog exposure | 1.03 (0.93 - 1.15) | 1.19 (1.07 - 1.33)** |
|  |  |  |
| *Assuming dog’s lifespan of 12 years in those with missing dog’s death data* | | |
| No dog exposure | Ref | Ref |
| Discontinued dog exposure | 0.78 (0.54 - 1.12) | 0.83 (0.58 - 1.20) |
| Continuous dog exposure | 0.99 (0.91 - 1.09) | 1.12 (1.02 - 1.23)* |
|  |  |  |

* P < 0.05, ** P < 0.01

# Table E16. Odds ratios of the association between dog exposure and high use SABA, assuming dog’s lifespan of 8 years and 12 years in those with missing dog’s death data

|  | Unadjusted OR (95% CI) | Adjusted OR (95% CI) |
| --- | --- | --- |
|  |  |  |
| *Assuming dog’s lifespan of 8 years in those with missing dog’s death data* | | |
| No dog exposure | Ref | Ref |
| Discontinued dog exposure | 1.62 (1.25 - 2.11)** | 1.52 (1.17 - 1.98)** |
| Continuous dog exposure | 1.16 (1.03 - 1.31)* | 1.21 (1.07 - 1.37)** |
|  |  |  |
| *Assuming dog’s lifespan of 12 years in those with missing dog’s death data* | | |
| No dog exposure | Ref | Ref |
| Discontinued dog exposure | 1.33 (0.93 - 1.92) | 1.32 (0.92 - 1.90) |
| Continuous dog exposure | 1.21 (1.10 - 1.33)** | 1.22 (1.11 - 1.35)** |
|  |  |  |

* P < 0.05, ** P < 0.01
